# Investigation of the Synergistic Effect of Brown Sugar, Longan, Ginger, and Jujube (Brown Sugar Longan Ginger Tea) on Antioxidation and Anti-Inflammation in *In Vitro* Models

**DOI:** 10.1155/2020/3596085

**Published:** 2020-12-02

**Authors:** Ping Lin, Kai-Wen Kan, Jia-Haur Chen, Yung-Kai Lin, Yung-Hao Lin, Yung-Hsiang Lin, Wei-Chun Hu, Chi-Fu Chiang, Chen-Meng Kuan

**Affiliations:** ^1^Research & Design Center, TCI CO., Ltd., Taipei 114, Taiwan; ^2^Research & Design Center, TCI Gene Inc., Taipei 114, Taiwan; ^3^Institute of Food Safety and Risk Management, National Taiwan Ocean University, Keelung City 202, Taiwan; ^4^Global Business Center, TCI CO., Ltd., Taipei 114, Taiwan

## Abstract

This research unveils the synergistic effect of brown sugar, longan, ginger, and jujube on the beneficial effects of antioxidation and anti-inflammation. Longan, ginger, and jujube are ubiquitous herbs in traditional Chinese medicine (TCM) and are frequently used in folk remedies. Longan and ginger have been reported to be beneficial for antioxidation, anti-inflammation, ant-obesity, and nonalcoholic fatty liver disease (NAFLD) improvements. However, the potential scientific and medical benefits of their combination Brown Sugar Longan Ginger Tea (BSLGT), a popular drink in Chinese cultures, are elusive. Through the *in vitro* methodologies, we discovered that BSLGT could significantly improve the mitochondrial activity, antioxidant capacity, lipid content, and inflammatory response in human hepatocytes. In addition, BSLGT also exerted positive effects on the downregulation of atherosclerosis-associated, vasoconstrictor, and thrombosis-related gene expression in human umbilical vein endothelial cells. In short, our experimental results successfully revealed that the antioxidative and anti-inflammatory effects of BSLGT may have the potential to improve liver metabolism and cardiovascular inflammation although solid evidence requires further investigation.

## 1. Introduction

Brown Sugar Longan Ginger Tea (BSLGT) is a popular folk beverage in Chinese societies, especially in winter. From the traditional Chinese medicine (TCM) point of view, BSLGT belongs to hot attributes and is beneficial to warm the body, expel cold, improve blood circulation, and mitigate the symptoms of the common cold (e.g., cough) [1, 2]. As indicated by its name, the main compositions of BSLGT contained brown sugar, longan, and ginger. Brown sugar is a ubiquitous additive in several Chinese medicine remedies considering its sweet taste and warm nature [1]. Longan (*Dimocarpus longan* Lour.) is a subtropical fruit and widely distributed in South and Southeast China [3]. Longan is rich in vitamins and minerals, and its bark, flower, seed, root, leaf, and fruit can be utilized for complex Chinese remedies [2]. Longan in TCM is considered a warm ingredient for nourishing blood and stabilizing spirit; thus, it is usually prescribed for hematopoiesis, as well as improvement in the nervous system, insomnia, stomach pain, and memory function [4]. Recent studies also discovered that longan was able to interfere with oxidative stress formation, inflammatory responses, the onset of cancer, and tyrosinase activity in cells thanks to its considerable amounts of polyphenolic compounds, such as gallic acid, corilagin, and ellagic acid [5–7]. Moreover, longan demonstrated the potential to amelioratemetabolic disorders (e.g., obesity) and impair the development of nonalcoholic fatty liver disease (NAFLD) [8]. Ginger was also an accessible seasoning herb in our daily lives. In ancient China (around 4^th^ BC), people already knew how to use ginger to treat various illnesses, such as stomach pain, diarrhea, and nausea [9]. In TCM, ginger was categorized as a Yan herbal, and it has the capabilities to reinforce the blood circulation and nourish the body [10]. Ginger possesses abundant polyphenolic compounds (e.g., gingerols, shagols, and zingerones), which confer high antioxidant, anti-inflammatory, and anticancer activities [11–13]. Clinical and rodent studies proved that ginger could increase insulin resistance, reduce blood lipid level, and stymie the onset of NAFLD [14–16]. In addition to the conventional ingredients of BSLGT, we incorporated jujube into the BSLGT. Jujube (*Ziziphus jujuba* Mill.) contains abundant bioactive compounds, such as vitamin C, phenolics, and flavonoids, and was used for the treatment of anorexia, fatigue, and anemia in TCM [17]. Not only does jujube enrich the flavor of the tea but also can reinforce the whole antioxidant capability.

Despite the fact that BSLGT was a handy drink, scientific evidence regarding its health benefits was still elusive. In this study, we attempted to use *in vitro* methodologies to assess its synergistic effects on the improvement of oxidative stress and inflammatory response, as a bridge for the studies of NAFLD and cardiovascular disease using HepG2 cells and human umbilical vein endothelial cells (HUVECs) to analyze the possible effects of BSLGT on NAFLD and cardiovascular disease. In this study, we found that BSLGT was able to significantly improve the mitochondrial activity, antioxidant capacity, lipid content, and inflammatory response in HepG2 cells and the vascular-related gene expression in HUVECs. As a result, BSLGT was possible to confer the prevention effects on the development of NAFLD and cardiovascular inflammation.

## 2. Materials and Methods

### 2.1. Materials

BSLGT was provided from Hangzhou Yosto Cosmetics CO., LTD., and the main ingredients of this product are brown sugar (10%), longan extract (1.87%), jujube extract (2.5%), and ginger extract (2.5%); minor ingredients (<0.2%) are Galangal extract, *Pueraria mirifica* extract, *Angelica dahurica* extract, and cinnamon extract; HepG2 (ATCC® HB-8065™), human umbilical vein endothelial cell (HUVEC; ATCC® CRL-1730™), cell culture medium (Dulbecco's modified Eagle's medium (DMEM, Gibco), 1% penicillin-streptomycin (Gibco), and 10% fetal bovine serum (FBS, Gibco)) were used [18]. MRS broth (BD Difco™), 2′,7′-dichlorofluorescin diacetate (DCFH-DA, Sigma), phosphate-buffered saline (PBS, Gibco), mitochondrial membrane potential detection kit (BD), interleukin-6 (IL-6) and tumor necrosis factor alpha (TNF-*α*) ELISA kits (Cloud-Clone Corp.), an RNA extraction kit (Genaid Biotech), and the nCounter® platform (NanoString Technologies) were used.

### 2.2. Mitochondrial Activity Assay

1 × 10^5^ HepG2 cells in 2 mL of culture media were added to each well of 6-well plates and incubated at 37°C for 24 hours. For sample treatment, the cells were treated with 0.5% or 1% BSLGT for 24 hours. Following the repeated PBS wash, the JC-1 Mitochondrial Membrane Potential Assay Kit used JC-1 dye to detect the mitochondrial membrane potential in a variety of cell types (Abcam, Cambridge, UK). The fluorescence signals were acquired by using a flow cytometer (excitation wavelengths: 450–490 nm; emission wavelengths: 510–550 nm) [19].

### 2.3. ROS Assay

1 × 10^5^ HepG2 cells in 2 mL of culture media were added to each well of 6-well plates and incubated at 37°C for 24 hours. For sample treatment, the cells were treated with 0.5% or 1% BSLGT for 24 hours. Following the repeated PBS wash, DCFH-DA (5 *μ*g/mL) dye solution coincubated with the cells for 15 minutes. Subsequently, the cells were exposed to 0.5 mM H_2_O_2_ for an hour. The fluorescence signals were acquired by using a flow cytometer (excitation wavelength: 450–490 nm; emission wavelengths: 510–550 nm) [20].

#### 2.3.1. Oil Red O Staining Assay

1 × 10^5^ HepG2 cells in 2 mL of the culture medium were incubated at 37°C in each well of 6-well plates for 18 hours. Subsequently, the cells were treated with 0.5% or 1% BSLGT in 2% FBS medium for 24 hours, and then, oleic oil (OA) was used to induce the formation of lipid droplets. Finally, the lipid accumulation results were evaluated by Oil Red O staining assay [21].

### 2.4. IL-6 and TNF-*α* Assays

1 × 10^5^ HUVECs were dispersed into each well of 6-well plates and incubated at 37°C for 24 hours. Subsequently, the cells were treated with 0.5% or 1% BSLGT for 24 hours. Following the repeated PBS wash, lipopolysaccharides (LPS, 5 *μ*g/mL) were used for immune stimulation. In the end, IL-6 and TNF-*α* levels were analyzed by using the ELISA kits [22].

### 2.5. Gene Expression Analysis

1.5 × 10^5^ HUVECs in 2 mL of the media with 1% BSLGT were coincubated for 24 hours. We collected the cells and extracted their total RNA by using the RNA extraction kit. 75 ng/*μ*L RNA extracts were used for the following expression analysis by using the nCounter® platform [23].

### 2.6. Statistical Analysis

The experimental results were analyzed by Student's *t*-test in Microsoft Excel software; *p* < 0.05 was considered a significant difference.

## 3. Results and Discussion

### 3.1. Antioxidant Effect

**Figure 1:**
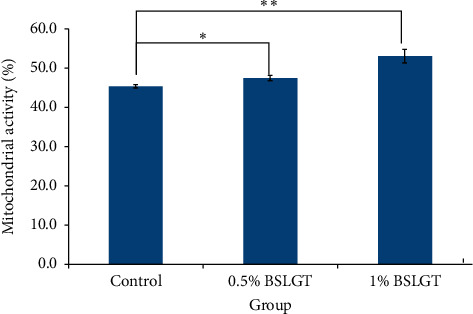
BSLGT treatment increased mitochondrial activity. HepG1 cells were treated with 0.5% and 1.0% of BSLGT for 24 hrs. JC-1 staining was performed for mitochondrial activity assay by flow cytometry. Also, statics results were performed by the *t*-test (*n* = 3; mean ± S.D.) (*∗*, *p* < 0.05; *∗∗*; *p* < 0.01). BSLGT: Brown Sugar Longan Ginger Tea.

**Figure 2:**
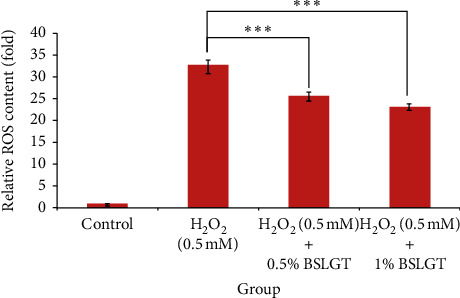
BSLGT pretreatment enhanced the antioxidation ability of HepG2. Different doses of BSLGT were pretreated with HepG2 for 24 hrs. Afterward, the H_2_O_2_ treatment was performed for inducing intracellular ROS levels. DCFH-DA staining was performed for ROS status measuring. Then, analysis was performed by flow cytometry. Also, statics results were performed by the *t*-test (*n* = 3; mean ± S.D.) (*∗∗∗*; *p* < 0.01 (corresponding to the H_2_O_2_ group)). BSLGT: Brown Sugar Longan Ginger Tea.

To explore whether BSLGT can regulate mitochondrial activity and antioxidant ability in the liver, BSLGT was used to treat HepG2 cells and examined by mitochondrial activity assay and ROS assay.  Figure 1 shows the results of the mitochondrial activities of HepG2 cells after treatment of 0.5% and 1% of BSLGT. As compared with the control group, 0.5% and 1% of BSLGT could increase the mitochondrial activities in HepG2 cells by 4.6% and 17%, respectively. The improvement of cell vitality exhibited a dose-dependent effect.  Figure 2 shows the results of the antioxidant ability of HepG2 cells after treatment of 0.5% and 1% of BSLGT. We used H_2_O_2_ to induce ROS expression, and we found that 0.5% and 1% of BSLGT reduced the ROS induction in HepG2 cells by 21.3% and 29.3%, respectively. Note that the improvement progress also corresponds to a dose-dependent trend. Some studies showed that longan extract increased the activity of mitochondrial H + -ATPase [24], and ginger extract promoted mitochondrial biogenesis and mitochondrial function via activation of the AMPK-PGC1ɑ signaling pathway both in mice and in HepG2 cells [25]. The longan pericarp exhibited radical scavenging activity, reducing activity, and liposome protection activity, as a natural antioxidant [4], and ginger extract can increase the gene expression of antioxidant enzymes and reduced the IL-1*β*-induced elevation of ROS [26].

### 3.2. Lipid Accumulation

**Figure 3:**
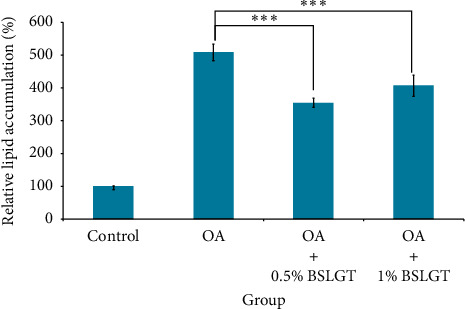
BSLGT pretreatment decreased the lipid accumulation of HepG2. OA was treated for inducing lipid droplet formation. Then the oil Red O staining assay was performed for lipid generation measurement. Also, statics results were performed by the *t*-test (*n* = 3; mean ± S.D.) (*∗∗∗*; *p* < 0.01 (corresponding to OA group)). BSLGT: Brown Sugar Longan Ginger Tea. OA: Oleic acid.

NAFLD was characterized by hepatic steatosis, steatohepatitis, and fibrosis [27]. Namely, the overwhelming lipid content in the liver incurs chronic inflammation and irreversible tissue damage. Thus, lipid elimination in the liver was imperative to prevent the development of NAFLD. To explore whether BSLGT can regulate lipid accumulation in the liver, BSLGT was used to treat HepG2 cells and examined by Oil Red O staining assay.  Figure 3 shows the lipid accumulation results of HepG2 cells after treatment of 0.5% and 1% of BSLGT. Compared to the OA group, 0.5% and 1% of BSLGT lowered the relative lipid contents of HepG2 cells by 30.3% and 20.1%, respectively. Some studies showed that longan flower water extract decreased hepatic lipids, inhibited lipase activity, sterol regulatory element-binding protein-1c (SREBP-1c), and fatty acid synthase (FAS) gene expressions [28]. The ginger supplementation decreased total cholesterol (TC) and triglyceride (TG) and inhibited liver steatosis by regulating the expressions of hepatic genes in HFD-induced mice model [29]. Moreover, dried jujube fruits reduced HFD-induced weight gain and central obesity and decreased abdominal and epididymal fat mass through the phosphoinositide 3-kinase (PI3K)-Akt pathway [30]. In this result, the improvement effect of lipid elimination may be attributed to the inhibition of adipogenesis and the improvement of lipolysis.

### 3.3. Downregulation of Inflammatory Response

**Figure 4:**
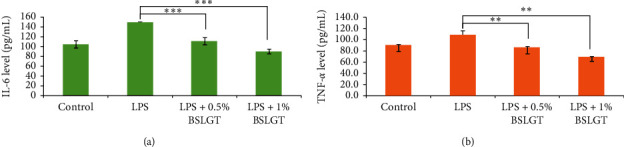
BSLGT pretreatment promoted the anti-inflammation ability of HepG2 by ELISA assay. LPS (5 *μ*g/mL) treatment was used for immune stimulation after BSLGT pretreatment. Then, ELISA was performed for immune response analysis. Also, statics results were performed by the *t*-test. (a) IL-6 detection result. (b) TNF-*α* detection results (*n* = 3; mean ± S.D.) (*∗∗*; *p* < 0.01; *∗∗∗*; *p* < 0.01 (corresponding to LPS group)). BSLGT: Brown Sugar Longan Ginger Tea. LPS: Lipopolysaccharides.

It had been proved that high levels of IL-6 and TNF-*α* were founded in patients with NAFLD [31]. Conversely, inhibition of IL-6 and TNF-*α* expression should improve the inflammatory response to the development of NAFLD. Thus, we want to know whether BSLGT can regulate IL-6 and TNF-*α* cytokines in the liver, using BSLGT to treat HepG2 cells, and examined by ELISA.  Figure 4 shows the improving results of the expression levels of IL-6 and TNF-*α* of HepG2 cells under the circumstance of BSLGT treatment. We used lipopolysaccharides (LPS) to induce inflammatory conditions. Compared to the LPS group, 0.5% and 1% of BSLGT downregulated the levels of IL-6 of HepG2 cells by 38% and 50%, respectively (Figure 4(a)) and the levels of TNF-*α* of HepG2 cells by 22% and 44%, respectively (Figure 4(b)). Some studies showed that longan seed extract reduced LPS-induced nitric oxide (NO), interleukin-1*β* (IL1*β*), IL-6, and COX2 productions and inhibited LPS activated c-Jun NH2-terminal protein kinase (JNK), extracellular signal-regulated kinases (ERKs), and p38 MAP kinases signaling pathways [32]. Ginger exerts suppressed adipose tissue inflammation (ATI) by inhibiting macrophage recruitment and downregulating proinflammatory cytokines [33]. In light of the outcomes of antioxidation, lipid content, and modulation of inflammation, BSLGT has the potential to hinder the development of NAFLD.

### 3.4. Improvement in Vascular Disease-Related Gene Expression

**Figure 5:**
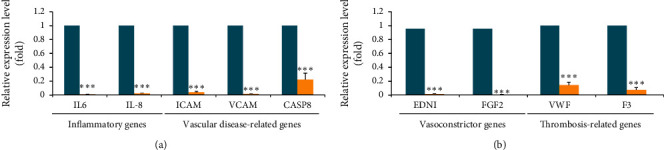
BSLGT treatment decreased inflammatory-, vasoconstrictor-, and thrombosis-associated gene expression of HUVECs. The gene expression profile was performed by qPCR analysis from the nCounter® platform. The blue bar indicated a control set gene expression level, and the yellow bar indicated 1% BSLGT-treated HUVECs gene expression level. Also, statics results were performed by the *t*-test (*n* = 3; mean ± *S*.D.) (*∗∗∗*; *p* < 0.001). (a) Inflammatory-associated genes: IL-6 (Interleukin-6), IL-8 (Interleukin-8), ICAM (Intercellular Adhesion Molecule), VCAM (Vascular cell adhesion protein), and CASP8 (caspase-8) (b) Vasoconstrictor- and thrombosis-related genes: EDN1(endothelin-1), FGF2 (fibroblast growth factor 2), VWF (von Willebrand factor), and F3 (coagulation factor III).

NAFLD was associated with impaired left ventricular diastolic dysfunction, alteration in energy metabolism, and disturbance of the cardiac rhythm [2]. Retrospective and prospective studies provided evidence of a strong association between NAFLD and the subclinical manifestation of atherosclerosis [2]. Finally, we want to explore whether BSLGT can regulate vascular disease-related gene expression in vascular endothelium, using BSLGT to treat HUVECs, and examined by gene expression analysis. 1% of BSLGT downregulated the inflammatory gene expression levels in HUVECs by the range of 4.5–114.1 folds (Figure 5(a)). Moreover, the 1% BSLGT also downregulated the vasoconstrictor gene expression levels (i.e., *endothelin-1* (*EDN1*) and *fibroblast growth factor 2* (*FGF2*)) by at least 63 folds and thrombosis-related gene expression levels (i.e., *von Willebrand factor* (*VWF*) and *coagulation factor III* (*F3*)) by at least 6.6 folds. Atherosclerosis was correlated with the IL-6- and IL-8-derived upregulation of vascular cell adhesion protein (VCAM) and intercellular adhesion molecule (ICAM) on endothelial cells, which result in monocytic cell adhesion and intimal thickening [27–29]. Expression of caspase-8 (CASP8) was correlated with the evaluated incidence rates of coronary events in patients [30]. EDN1 and FGF2 were involved in the modulation of vascular tone in the human body, and their overexpression may instigate hypertension [31, 32]. On the other hand, the high expression of VWF and F3 raised the risk of thrombosis [34]. In addition, some studies showed that polyphenolic extract from longan can generate new blood vessels and capillaries, regenerating new dermal tissue and remodeling the newly formed tissue [35]. The ginger crude extract increased vasoprotection via the suppression of nitric oxide synthase and cyclooxygenase [36]. Gingerol, a pungent ingredient of ginger, inhibited VEGF-induced proliferation of human endothelial cells and caused cell cycle arrest in the G1 phase and the formation of the new blood vessels in the mouse cornea [37]. Hence, BSLGT might substantially improve the status of the cardiovascular system.

## 4. Conclusions

In summary, this work successfully demonstrated the synergistic effect of brown sugar, longan, ginger, and jujube for antioxidation and anti-inflammation. BSLGT noticeably improved the mitochondrial activity and antioxidant capacity, as well as reduced lipid accumulation in human hepatocytes. More importantly, it also conferred the remarkable inhibition effect on the critical inflammatory response to the development of NAFLD. To verify the legitimacy of the availability of BSLGT for improvement of cardiovascular function, we also investigated the relationship between BSLGT and vein endothelial cells. BSLGT could downregulate the atherosclerosis-associated, vasoconstrictor, and thrombosis-related gene expression in human umbilical vein endothelial cells, which sustained that BSLGT may be possible to improve cardiovascular function. Based on these encouraging results, BSLGT has the potential for the prevention of chronic liver and cardiovascular diseases although solid evidence requires further investigation.

## Data Availability

All data (figures) in this article are available from the authors for free by the e-mail requirement. The authors do not prefer to upload the image results on another third party considering their further business concern.
